# Running-related injuries and risks among runners in township running clubs in Soweto, Johannesburg

**DOI:** 10.17159/2078-516X/2026/v38i1a20618

**Published:** 2026-06-15

**Authors:** V Masilana, S Kunene

**Affiliations:** 1 Physiotherapy Department, Faculty of Health Sciences, University of the Witwatersrand, Johannesburg, South Africa; 2 Wits Sports and Health, Faculty of Health Sciences, University of the Witwatersrand, Johannesburg, South Africa

**Keywords:** Running-related injuries, risk factors, township runners, injury prevalence

## Abstract

**Background:**

The growing popularity of running has led to a rise in running-related injuries (RRIs), with runners in under-resourced communities facing heightened risks.

**Objectives:**

This study aimed to determine the prevalence and risk factors associated with RRIs among recreational and advanced runners in a township area of South Africa.

**Methods:**

A cross-sectional study design was employed, involving runners from six running clubs in Soweto township (south of Johannesburg, South Africa). Data were collected using a self-administered questionnaire via the RedCap online platform and analysed using STATA software. Both descriptive and inferential statistics were generated.

**Results:**

A total of 84 runners participated, comprising 51% females (n = 43) and 49% males (n = 41). The overall prevalence of RRIs was high (63%, n = 53), with a slightly higher injury rate among females (51%, n = 27) compared to males (49%, n = 26). Age (runners over 25 years) (odds = 1.11, p = 0.004), increased running load (particularly among medium and long distances) (odds = 1.47, p = 0.053; and odds = 2.65, p = 0.021, respectively), and running terrain (varied running inclines) (odds = 1.42, p-value = 0.032) were identified as significant risk factors.

**Conclusion:**

The study revealed a high prevalence of RRIs and identified multiple risk factors. These findings underscore the need for targeted intervention studies focusing on injury prevention and rehabilitation to reduce the burden of RRIs.

Running is widely recognised as the most popular form of physical activity, with an estimated 225 million people participating regularly in running or jogging worldwide.^[1]^ This widespread engagement is largely due to the numerous health benefits associated with running. According to Loprinzi and Cardinal, running contributes significantly to public health by reducing the risk of noncommunicable diseases, aiding in weight management, alleviating pain, enhancing physical function, preventing falls, improving cognitive performance, promoting better sleep quality, lowering fracture risk, and supporting mental health and overall well-being.^[2]^

As the fitness industry continues to evolve, more people are becoming involved in physical activities, particularly running. Between 2001 and 2016, global participation in running increased by 17%, reflecting a growing awareness of its health advantages.^[3]^ However, this rise in popularity has also led to an increase in running-related injuries (RRIs), posing significant challenges for runners worldwide.

Studies indicate that approximately 74% of runners sustain injuries in a single year.^[4]^ A systematic review by Kakouris, Yener, and Fing identified the most prevalent RRIs: patellofemoral pain (runner’s knee) (17%), medial tibial stress syndrome (shin splints) (9%), plantar fasciitis (8%), iliotibial band syndrome (ITB) (8%), and Achilles tendinopathy (7%).^[5]^ Among ultramarathoners, the ankle (35%), knee (28%), and lower leg (13%) are the most frequently injured anatomical sites. These injuries not only affect runners’ physical health but also hinder their ability to engage in occupational, recreational, and sporting activities. Beyond the physical toll, RRIs can lead to substantial economic consequences. The increasing popularity of running is also evident in sub-Saharan Africa, where participation has mirrored global trends. Countries such as Kenya, Ethiopia, and South Africa have produced some of the world’s elite long-distance runners, and running is deeply embedded in the cultural fabric of many African societies.

Despite this rich tradition, runners in sub-Saharan Africa are not immune to RRIs. For instance, a Kenyan study found that 63% prevalence of running injuries, highlighting the widespread nature of these injuries even in regions with strong running cultures.^[6]^ This finding aligns with broader concerns raised by Loprinzi and Cardinal, who emphasise that the health benefits of running can only be sustained through effective injury prevention and management strategies. These findings underscore the need for targeted interventions.^[2]^

In South Africa, promoting physical activity such as jogging has become a public health priority, particularly in densely populated areas. However, runners in these communities often face unique challenges, including limited access to coaching, training resources, and healthcare services.^[7]^ These constraints can significantly elevate the risk of RRIs.

The literature identifies a range of intrinsic and extrinsic risk factors for running injuries. Intrinsic factors include malalignment of body parts, muscle imbalances, muscle weakness, limited flexibility, leg-length discrepancies, previous injuries, and body composition.^[7]^ Extrinsic factors encompass training errors, unsuitable running surfaces, inappropriate footwear, environmental conditions, psychological stressors, and poor nutrition. Injuries typically occur when the physical load of running exceeds the body’s capacity to manage it. Therefore, it is essential for runners to enhance their physical resilience to meet the demands of their training loads.

South African runners participate in various events, with marathons and ultramarathons being particularly popular. The growing interest in running among individuals from low socioeconomic backgrounds highlights the need for inclusive support systems. Unfortunately, the lack of comprehensive research on RRIs in South Africa continues to hinder efforts to promote safe and effective running practices.

This study seeks to address this gap by investigating the prevalence of RRIs and identifying associated risk factors among runners in the Soweto community of South Africa. The findings aim to inform targeted interventions that can enhance runner safety and performance in township-based running clubs.

## Methods

### Study design

This study employed a cross-sectional design to investigate RRIs and associated risk factors among runners in Soweto.

### Participants and sampling

Participants included male and female runners aged 18–60 years, recruited from six athletic clubs in Soweto, a township within the City of Johannesburg Municipality, Gauteng Province, South Africa.

A purposive sampling technique was used to recruit 84 participants from a pool of 107 potential candidates. Since the study aimed to determine the prevalence and risk factors of RRIs, it was vital to include individuals with direct experience of the running environment. This non-probability sampling method allowed for the deliberate selection of participants based on specific inclusion criteria, ensuring representation across relevant subgroups within the target population. Purposive sampling was especially appropriate for this research due to recruitment difficulties and the need for targeted insights. It enabled efficient access to a diverse yet pertinent group of runners via an online platform, supporting a focused and detailed examination of the research questions while maintaining diversity within the sample. The sample size was calculated using the Raosoft statistical tool, with parameters set at a 95% confidence level and a 5% margin of error.

### Data collection tools

Data were collected using a self-administered questionnaire developed, validated, and structured into four sections. Section A collected demographic information, including height, weight, hand dominance, gender, race, and age. Section B focused on participants’ sports backgrounds, including running style, level of participation, and years of experience. Section C addressed the frequency and treatment history of RRIs across various anatomical regions, including the foot, ankle, shin, knee, thigh, hip, groin, buttocks, iliotibial band (ITB), and lower back. Section D explored risk factors associated with RRIs, including running load, participation frequency, environmental influences, equipment used, and running methodologies.

To ensure the reliability and validity of our online questionnaire assessing running injury prevalence and associated risk factors, a two-step process was followed. For content validity, the questionnaire items were developed based on existing literature and expert input from sports physiotherapists. A panel of experts reviewed the questionnaire to assess the relevance, clarity, and comprehensiveness of each item, ensuring alignment with the study objectives.

To ensure reliability, a pilot study was conducted with a sample of nine from the target population. No modifications were required following the pilot. Unfortunately, test-retest reliability could not be evaluated.

### Data collection procedure

Participants were contacted via phone, email, and in-person meetings. The study’s purpose, procedures, and voluntary nature were explained, and informed consent was obtained. The questionnaire was administered via the RedCap online platform, with the link distributed through email and WhatsApp by club coaches and managers. Weekly reminders were sent to encourage timely completion. Each questionnaire took approximately 15–20 minutes to complete.

### Statistical analysis

Data analysis was performed using STATA (by StataCorp LLC: https://www.stata.com/company/), a licensed statistical software chosen for its reproducibility and code-based analysis capabilities. Descriptive statistics were used to summarise demographic data, sports history, and injury prevalence through frequencies and percentages.

Data distribution was checked early after data cleaning and before selecting the statistical test. Univariate analysis was conducted to examine associations between individual risk factors and RRIs. Inferential statistics, including Pearson’s chi-square tests, odds ratios (OR), and logistic regression, were used to determine associated risk factors. The level of significance was set at *p* = 0.05.

### Ethical considerations

Ethical clearance was obtained from the Human Research Ethics Committee (HREC) at the University of Witwatersrand (Clearance number: M220234). Permission to conduct the study was also granted by Central Gauteng Athletics (CGA), the governing body for athletics in Soweto.

Participants were not exposed to any risk. Each participant received an information sheet detailing the study and signed a consent form prior to data collection.

Initially, the response rate was low (33%). To improve participation, reminders were sent via email, WhatsApp, and SMS. The questionnaire remained accessible for two weeks, allowing participants to complete it at their convenience.

## Results

### Demographic characteristics of the participants

Eighty-four runners out of 107 participated in the study (79% response rate). Of these, 43 (51%) were female and 41 (49%) were male (Table 1). Twenty-three participants (27%) were younger than 25 years, while 61 (73%) were older than 25. A greater proportion of female runners (37%, n = 31) identified as beginner runners compared to their male counterparts (14%, n = 12). The majority of participants (40%, n = 34) had a Body Mass Index (BMI) within the normal range. No significant associations were found between demographic characteristics and level of participation.

### Sports-specific characteristics of participants

Table 2 illustrates notable differences in running preferences between beginner and advanced runners. Beginner runners were defined as individuals who were new to running or returning after a prolonged break (having run for less than six months), whereas advanced runners were those who had been running consistently for at least two years. A substantial proportion of beginner runners (59%, n = 50) reported frequent participation in short-distance running, whereas only 10% (n = 8) of advanced runners engaged in short-distance events. Conversely, long-distance running was more common among advanced runners, with 20% (n = 17) reporting participation, compared with just 11% (n = 9) among beginners. These differences were statistically significant, as confirmed by a chi-square test (p = 0.001*).

Training frequency also varied significantly between the two groups. Most beginner runners (43%, n = 36) preferred running two to three times per week, while only 6% (n = 5) of advanced runners followed this schedule. Notably, 18% (n = 15) of advanced runners trained four to five days per week and beginners 14% (n = 12). These variations in training frequency were statistically significant (p = 0.002*).

Regarding participation in other sports, 22% (n = 19) of beginner runners reported involvement in additional athletic activities. However, the difference in cross-sport participation between beginner and advanced runners was statistically significant (p = 0.021).

A total of 63% (n = 53), more than beginners (36%, n = 30), reported having sustained RRIs. These results showed a significant difference (p = 0.041). In terms of injury management, 17% (n = 9) of beginner runners and 17% (n = 9) of advanced runners reported not seeking medical advice for their injuries, suggesting a potential gap in injury care across both groups.

### Prevalence and characteristics of RRIs

An analysis of the data by gender revealed no significant variation in the overall prevalence of RRIs, which was estimated at 63% (n = 53). Among those affected, 51% were female and 49% were male (Table 3).

Within the injured group, beginner runners exhibited a higher prevalence of RRIs (58.5%, n = 31) compared to advanced runners (42%, n = 22) (Table 3). Additionally, individuals who exclusively participated in running appeared more susceptible to RRIs (53%, n = 28) than those who engaged in both running and other sports.

Training frequency also influenced injury prevalence. Runners who trained two to three days per week reported a higher incidence of injuries (43%, n = 23), suggesting that moderate training frequency may be associated with increased vulnerability to RRIs.

Knee injuries (72%) were more common compared to other types of injuries (ankle: 53%; foot: 38%; other: 38%) (Table 3).

### Risk factors for RRIs

The analysis of socio-demographic and environmental factors revealed that the frequency of running days per week significantly influenced the likelihood of sustaining RRIs (Table 4). Female runners appeared to be at a lower risk of RRIs compared to male runners, with an odds ratio of 0.25 (p = 0.103), although this result did not reach statistical significance.

Participants who ran four to five times per week were nearly twice as likely to experience RRIs (odds ratio = 1.87; p = 0.021), indicating a statistically significant association between increased training frequency and injury risk.

Additionally, a notable correlation was observed between participation in other sports alongside running and the risk of RRIs (p = 0.08). Specifically, individuals who did not engage in other sports had a 0.35 times lower chance of developing RRIs, suggesting that cross-training may contribute to injury prevention.

Participants aged 25 years and older demonstrated a higher risk of RRIs, with odds 1.11 times greater than those aged 25 years and younger (p = 0.004) (Table 4). Additionally, running longer distances, specifically greater than 10 kilometres, was significantly associated with increased injury risk, with an odds ratio of 2.65 (p = 0.021).

Surface type also influenced injury likelihood. Runners who trained on varied-level surfaces were nearly 50% more likely to sustain RRIs (odds ratio = 1.42; p = 0.032) compared to those who ran on flat surfaces, which served as the reference group.

Although running with older shoes appeared to elevate the risk of RRIs by 1.43 times compared to using modern shoes, this association did not reach statistical significance (p = 0.072). Similarly, no meaningful difference in injury risk was observed between runners with left-leg dominance (reference group) and those with right-leg dominance (odds ratio = 0.88, p = 0.430).

Overall, female athletes were less likely than their male counterparts to sustain RRIs (Table 5). This trend was statistically significant among novice runners, with females showing reduced odds of injury (odds ratio = 0.75; p = 0.05). Age remained a significant explanatory factor for RRIs, particularly among beginner-level runners aged 25 or older, who had a higher risk (odds ratio = 1.56; p = 0.031) than advanced runners (odds ratio = 1.09; p = 0.000). Notably, age did not substantially increase the risk of RRIs among advanced runners.

Distance emerged as a significant risk factor among advanced athletes. Statistically significantly elevated risks were observed for both medium-distance (odds ratio = 2.07; p = 0.050) and long-distance running (odds ratio = 3.01; p = 0.032). Among novice runners, training two to three times per week increased the risk of RRIs by 1.56-fold (p = 0.011), which was 0.44-fold lower than the risk associated with the same training frequency among advanced runners. In contrast, advanced runners training four to five days per week had nearly double the risk of RRIs (odds ratio = 1.97; p = 0.010), exceeding the risk observed in beginner runners with the same schedule (odds ratio = 1.56; p = 0.231).

Interestingly, non-participation in other sports did not significantly influence the risk of RRIs among beginner runners (odds ratio = 1.10; p = 0.110). However, among advanced runners, participation in other sports was associated with an increased risk of RRIs (odds ratio = 0.77; p = 0.050), suggesting that cross-training may not always be protective at higher competitive levels.

For beginner runners, running on varied terrain was associated with a 75% increased risk of RRIs (odds ratio = 1.76; p = 0.006) compared to running on flat surfaces.

## Discussion

This study revealed a gender-based distinction in running participation: advanced running was predominantly male, while recreational running was largely female. One plausible explanation for the higher proportion of female recreational runners is the emphasis on enjoyment and health-promoting behaviours, which aligns with previous findings that women are more likely than men to engage in physical activity for health benefits.^[8]^ This trend seems inconsistent with global patterns, including findings from South African recreational running communities, where women participate less than men in organised running events for wellness and social engagement.^[9]^

The majority of runners in both advanced and novice categories were over 25 years old. Notably, a larger proportion of runners under 25 were found in the novice group, supporting existing literature. Individuals over 25 may experience greater personal and professional stability, allowing more time for leisure activities such as running.^[10]^ This age-related trend may also reflect accumulated experience and motivation among older recreational runners, encouraging sustained participation and peer influence.^[10]^ In African contexts, such as Kenya, age and experience are often linked to endurance development and long-distance running success, particularly in advanced training camps.^[6,11]^

Sport-specific characteristics showed that novice runners mainly participated in short-distance races, while advanced runners preferred long-distance events. This tendency among advanced runners might be due to improved endurance and training experience, consistent with previous research indicating that advanced athletes tend to choose more challenging distances to enhance skills and stamina. Novice runners often focus on building basic fitness and gradually moving to longer distances, whereas advanced runners aim for competitive goals and personal bests. Haugen highlighted the considerable time and effort needed for long-distance training, a point supported by East African advanced training environments where high mileage and altitude adaptation are common.

Training frequency also differed between groups. Most novice runners trained 2–3 times per week, while advanced runners typically trained 4–5 times or more. This disparity reflects differences in training intensity and volume, with advanced runners adhering to rigorous regimens to enhance performance.^12^ Novices, in contrast, often focus on gradual adaptation and injury prevention. Recovery and injury risk management are particularly crucial for beginners, who may lack the physical conditioning to withstand frequent training loads.

The study found a high overall prevalence of RRIs, with no significant gender differences. This contrasts with studies suggesting higher injury susceptibility among female runners. For instance, Van der Worp et al. reported that women were 2.2 times more likely to develop patellofemoral pain syndrome. Anatomical and biomechanical differences may contribute to this disparity.^[13]^

Older runners (over 25) had a slightly higher prevalence of RRI, suggesting age as a potential risk factor. El-Metawally et al. found that injury risk increased by 1.3 times per additional year of age.^[14]^ However, the small difference between age groups implies that other variables may play a more significant role. Lopes et al. noted that older long-distance runners face greater injury risks, while Alerntorn-Geli et al. observed a reduced injury risk among athletes over 50.^[15,16]^ These mixed findings highlight the need for further research, particularly in African populations where age-related physiological and environmental factors may differ.

Long-distance runners in this study had a high prevalence of RRI, indicating a higher risk among experienced runners. Novices may be more cautious and less likely to overexert themselves.^[17]^ The RRI prevalence also increased with training frequency: 77% among those running 4–5 days per week and 100% among daily runners. This trend suggests that overuse injuries may result from inadequate recovery and repetitive stress. Common injuries such as shin splints are often linked to high training frequency and insufficient biomechanical adaptation. Similar patterns have been observed in African endurance runners, in which high training loads without adequate recovery increase injury risk.^[6,7,11]^

Advanced runners experienced significantly more RRIs than novices, likely due to the physical demands of high-intensity training and competition. These regimens can lead to overuse injuries, muscle strains, and joint issues, which hinder performance and long-term musculoskeletal health.^[18]^ Overuse injuries often result from training loads that exceed the tolerance of musculoskeletal structures. Muscle strains typically occur during eccentric contractions or fatigue, while joint injuries may stem from repetitive loading or biomechanical inefficiencies. Biomechanical analysis is essential for understanding injury mechanisms, particularly in advanced African runners, where gait patterns and terrain variability may influence injury profiles.^[19]^

The running surface also influenced RRI prevalence. Participants running on wet surfaces reported more injuries than those on dry surfaces, likely due to reduced traction and stability. This finding aligns with the current literature, which shows that non-uniform running surfaces, including wet surfaces, increase the risk of sprains and falls, especially on uneven or slippery terrain.^[7,20]^

In their study, Ferro-Sánchez et al. demonstrated that running performance and biomechanics are significantly influenced by the type of surface on which running occurs.^[20]^ Variations in running surfaces can alter an athlete’s technique and contribute to an elevated risk of musculoskeletal injuries. Given the substantial role that surface conditions play in injury incidence, it is essential that runners receive targeted training focused on injury prevention strategies. While avoiding unsuitable running surfaces is one preventive measure, such avoidance is not always feasible, particularly in competitive race settings. Therefore, runners must be equipped with the necessary skills and tactical knowledge to adapt effectively and safely to diverse surface conditions.

## Conclusion

This study highlights a notably high prevalence of RRIs and identifies a range of contributing risk factors across different levels of athletic experience. The findings offer valuable insights for developing targeted injury prevention and management strategies, particularly by distinguishing the nuanced injury patterns between novice and advanced runners. For beginners, the increased risk associated with varied terrain underscores the need for gradual exposure and terrain-specific training. Conversely, for advanced athletes, the complex relationship between sports engagement and injury risk calls for more sophisticated monitoring and support systems.

Importantly, the study recommends personalised treatment approaches that reflect the diverse nature of RRIs and the changing needs of runners throughout their careers. These insights have implications not only for clinical practice but also for policy development and future research paths.

In addition to biomechanical and training-related factors, the integration of rest and recovery protocols, cross-training, nutrition, hydration, and the use of monitoring tools, currently underutilised in some training regimens, should be emphasised in injury prevention guidelines. Within the South African context, particularly in communities such as Soweto, the development of these guidelines could benefit from a collaborative effort involving sports scientists, coaches, and medical professionals specialising in RRIs.

Furthermore, future research should explore the role of physiotherapy in preventing RRIs in this unique population, while accounting for prevalent comorbidities and socio-environmental factors. Such investigations could contribute to more inclusive and context-sensitive approaches to injury prevention and athlete care.

## Figures and Tables

**Table 1:** Demographic characteristics of participants (n = 84)

	Beginner %(n)	Advanced %(n)	Total %(n)	Chi^2^ p-value

**Gender**				

Male	32%(27)	17%(14)	49%(41)	0.53
Female	37%(31)	14%(12)	51%(43)	

**Age**				

25 and below	20%(17)	7%(6)	27%(23)	0.55
Over 25 Years	49%(41)	24%(20)	73%(61)	

**Body mass index**				

Underweight	10%(8)	2%(2)	12%(10)	0.81
Normal	27%(23)	13%(11)	40%(34)	
Overweight	14%(12)	8%(7)	23%(19)	
Obese	18%(15)	7%(6)	25%(21)	

**Table 2:** Sports-specific characteristics of participants (n=84)

	Beginner %(n)	Advanced %(n)	Chi^2^ p-value

**Type of running**			

Long Distance	11%(9)	20%(17)	0.001*
Short Distance	59%(50)	10%(8)	

**Average number of days run per week**			

0–1 Day/week	14%(12)	5%(4)	0.002*
2–3 days/week	43%(36)	6%(5)	
4–5 days/week	14%(12)	18%(15)	

**Participation in other sports**			

Yes	46%(39)	16%(13)	0.021*
No	22%(19)	16%(13)	

**Type of running surface**			

Natural surfaces	7%(6)	4%(3)	0.445*
Man made surfaces	67%(56)	22%(19)	

**Running incline type**			

Flat Surface	31%(26)	14%(12)	0.635
Varied Surface	37%(31)	18%(15)	

**Temperature conditions**			

Cold	7%(6)	4%(3)	0.870
Hot	62%(52)	27%(23)	

**Sustained injuries previously**			

No	32%(27)	5%(4)	0.041*
Yes	36%(30)	27%(23)	

**Consulting for injuries (n = 53)**			

No	17%(9)	17%(9)	0.368
Yes	42%(22)	24%(13)	

* indicates significance level p < 0.05

**Table 3:** Breakdown of the injury prevalence according to types (n=53)

	Prevalence	Knee	Ankle	Foot	Other

**Gender**					

Female	51% (27)	37% (10)	26% (7)	19% (5)	19% (5)
Male	49% (26)	35% (9)	27% (7)	19% (5)	19% (5)

**Competitive level**					

Beginner	59% (31)	42% (13)	26% (8)	16% (5)	16% (5)
Advanced	42% (22)	41% (9)	14% (3)	14% (3)	32% (7)

**Participation in other sports**					

Yes	47% (25)	28% (7)	32% (8)	12% (3)	28% (7)
No	53% (28)	36% (10)	18% (5)	14% (4)	32% (9)

**Average number of days run per week**					

>5 days/week	17% (9)	56% (5)	22% (2)	22% (2)	0
4–5 days/week	32% (17)	29% (5)	12% (2)	12% (2)	47% (8)
2–3 days/week	43% (23)	26% (6)	26% (6)	22% (5)	26% (6)
Once a week	8% (4)	50% (2)	50% (2)	0	0

Data expressed as percentage % (n).

**Table 4:** Risk factors for running-related injuries

	Odds ratio	p-value	95% Confidence interval

**Gender**			

Male [reference]			
Female	0.25	0.103	0.175–10.193

**Age**			

25 Years and Below [Reference]			
Over 25 Years	1.11*	0.004	1.001 – 1.332

**Average number of days run per week**			

0–1 Day/week [reference]			
2–3 Days/Week	1.75*	0.039	1.201 – 1.930
4–5 Days/Week	1.87*	0.029	1.870 – 2.251
More Than 5/Week	2.35	0.234	0.992 – 5.473

**Temperature conditions**			

Cold [reference]			
Hot	1.25	0.517	0.651 – 2.810

**Type of running surface**			

Natural surfaces [reference]			
Man made surfaces	0.47	0.829	0.142 – 3.187

**Participate in other sports**			

Yes [reference]			
No	0.35*	0.008	0.272 – 0.865

**Body mass index (BMI)**			

Underweight [reference]			
Normal	1.02	0.072	0.913 – 3.621
Overweight	1.01	0.074	1.012 – 2.651
Obese	0.97	0.231	0.822 – 1.932

**Types of running**			

Short distance [reference]			
Medium Distance	1.47*	0.053	1.376 – 2.124
Long Distance	2.65*	0.021	1.953 – 3.210

**Leg dominance**			

Left [reference]			
Right	0.88	0.430	0.765 – 2.345

**Shoe conditions**			

New [reference]			
Old	1.43	0.070	1.002 – 2.345

**Running incline type**			

Flat surface [reference]			
Varied surface	1.42*	0.032	1.130 – 3.445

**Level of competition**			

Beginner [reference]			
Advanced	2.20*	0.023	1.750 – 3.410

* Indicates statistically significant association (p < 0.05)

[reference] indicates the reference value used to determine odds ratio

**Table 5:** Risk factors for running-related injuries (beginner vs advanced)

	Beginner		Advanced	

	Odds ratio	p-value	Odds ratio	p-value

**Gender**				

Male [reference]				
Female	0.75*	0.05	0.50	0.342

**Age**				

25 years and below [reference]				
Over 25 years	1.56*	0.03	1.09	0.001

**Type of running**				

Short distance [reference]				
Medium distance	1.06	0.43	2.07	0.050
Long distance	***	***	3.01	0.032

**Average number of days per week**				

1 day/week [reference]				
2–3 days/week	1.5*	0.01	1.12	0.050
4–5 days/week	1.56	0.23	1.97	0.011
More than 5 days/week	***	***	1.26	0.043

**Participate in other sports**				

Yes [reference]				
No	1.1	0.11	0.77	0.050

**Running incline**				

Flat surface [reference]				
Varied surface	1.76	0.06	1.05	0.020

* Indicates statistically significant association (p < 0.05),

*** indicates “Not applicable”.

[reference] indicates the reference value used to determine odds ratio.
